# Parents, health professionals and footwear stakeholders’ beliefs on the importance of different features of young children’s footwear: a qualitative study

**DOI:** 10.1186/s13047-022-00580-1

**Published:** 2022-10-12

**Authors:** Cylie M. Williams, Helen A. Banwell, Kade L. Paterson, Katherine Gobbi, Sam Burton, Matthew Hill, Emma Harber, Stewart C. Morrison

**Affiliations:** 1 School of Primary and Allied Health Care, 47-49 Moorooduc Hwy, Monash University, Frankston, Victoria 3199 Australia; 2 Centre for Biomechanics and Rehabilitation Technologies, Staffordshire University, Staffordshire, UK; 3 Allied Health and Human Performance, University of South Australia, Adelaide, Australia; 4 Centre for Health, Exercise and Sports Medicine, The University of Melbourne, Melbourne, Victoria Australia; 5 Parent (Consumer Representative), Victoria, Australia; 6 Bobux International, Auckland, New Zealand; 7 Parent (Consumer Representative), Church Stretton, UK; 8 School of Life Course and Population Sciences, King’s College, London, UK

**Keywords:** Shoes, Children, Gait, Qualitative, Foot, Footwear

## Abstract

**Background:**

A small but building pool of evidence of the impact of footwear on children’s function means understanding the different beliefs of stakeholders about footwear key features and flexibility is critical for translation into recommendations and to support parents and caregivers in purchasing footwear for their children. Therefore, this research aimed to describe how different stakeholders (health professionals, parents, and footwear industry representatives) described the importance of flexibility and other footwear features for young children.

**Methods:**

This qualitative study was nested within an international modified Delphi online survey. Participants responded to open-ended questions about footwear component flexibility and asked if and why flexibility in these areas were important. Participants also described any other important footwear features. Inductive thematic analysis was used to generate themes.

**Results:**

There were 121 responses from three stakeholder groups including health professionals (*n* = 90), parents of young children (*n* = 26) and footwear industry representatives (*n* = 5). Overarching themes described by participants included developmental impacts of footwear, therapeutic impact and how footwear may play a role in function.

**Conclusion:**

There were key differences in how stakeholders viewed footwear and any perceived benefits of footwear components, much of which was not backed with empirical evidence. It was also identified that health professionals are using footwear within treatment recommendations. This work highlights the importance of understanding circumstances in which footwear may have a therapeutic impact or be the first line of treatment for children with complex foot needs. This is the first step in developing contemporary footwear recommendations for parents and caregivers.

## Background

The purchase of footwear can be a concern for many parents and caregivers. They often hold beliefs that incorrect footwear may negatively impact on the child’s foot, either in terms of morphology or function [1]. The promotion of clear and credible footwear information is therefore important to help parents and caregivers with footwear purchasing decisions. Ideally, footwear recommendations should be accessible (understood by parents), translatable (parents need to understand what recommendations mean when purchasing) and realistic (reflecting the shifts in purchasing habits) [1–3]. Interestingly, footwear advice is plentiful, with recommendations promoted by the footwear industry through advertising, professional bodies [4], government [5], specialist clinical groups [6], and parent [online] communities [7]. The resources transcend media and platforms, but commercially produced resources appear to be the most accessible. However, recent work has identified issues with the clarity of these recommendations (and their validity) [3], which feeds into a broader narrative about footwear literacy and parental concern [1].

Footwear recommendations for children who have typically developing feet and gait appear to have evaded scrutiny in scientific literature, with the majority of recommendations being historical and author- or profession group-developed [2, 8, 9]. As such, there has been little shift in the quality and robustness of the information being promoted by stakeholders. Recommendations for desirable footwear characteristics are not static across childhood and as foot demands change. This means any recommendations should be tailored and specific. This is particularly pertinent to the first 6 years, where a child’s development and foot growth and change is the greatest. There is growing scrutiny of footwear practices and an evolving momentum around footwear use [10, 11] which is challenging key foundations of footwear advice. Despite this, the importance of contemporary recommendations for footwear purchases should not be overlooked and reconceptualising these recommendations must involve all stakeholders.

Footwear research with children under the age of six, or young children, has focused primarily on footwear design components such as sole flexibility [12]. This is because there is rapid foot change thought to be associated with growth and tissue plasticity in the first 6 years [12] and a theoretical shift to wanting feet to develop in a ‘natural’ way [13]. There is no evidence to support or refute this hypothesis, nor whether influencing foot movement is positive or negative. This means the wider perceptions of stakeholders, such as health professionals, and footwear industry professionals are useful to help inform future research in this field. Furthermore, parents and caregivers’ describe the most concern when purchasing footwear for their children during these early years [1]. Researchers have recently also explored how young children describe comfort [14], when young children wear footwear with different features, and how these features are described [15]. Children’s footwear research has also focused on which footwear features may be therapeutically important if a child has a foot, leg or gait problem [16]. This small but building pool of evidence of the impact of footwear’s on functional abilities mean understanding the different beliefs of stakeholders about footwear flexibility and key features are critical for research translation. Initiatives to harmonise footwear information ensure robust recommendations for all stakeholders are needed given the diversity of stakeholders who provide and receive information.

The purpose of this work is to contribute to a contemporary footwear dialogue and support further work which translates outcomes into enhanced footwear literacy for children and their parents. The primary aim of this nested study was to describe how different stakeholders (parents, health professionals and footwear industry representatives) viewed the importance of flexibility of key footwear components. Our secondary aim was to understand what other footwear features stakeholders viewed as important for young children.

## Methods

### Design

This study was nested within an international three-round modified Delphi online survey. The methodology and results of the larger study resulted in development of a young children’s footwear taxonomy and are published elsewhere [15]. The present study used data collected through open ended questions asked of participants in the first round of the Delphi study. Reporting this research was guided by the COnsolidated criteria for REporting Qualitative research (COREQ) checklist where applicable [17].

### Participants and setting

Three groups of stakeholders were invited to respond. These stakeholders were: a) parents of children under the age of 6 years who had purchased shoes for their child in a shoe store with fitting support. (Parent stakeholders), b) health professionals who had made regular footwear recommendations for children under the age of 6 years, in the past 6 months (Health professional stakeholders) and c) professionals who had sold footwear in the past 6 months, for children under the age of 6 years or researchers who had researched young children’s footwear in the past 10 years (footwear industry stakeholders).

Recruitment advertisements were visually customised to each group. The survey was open to participants in any country, and this was stated in advertisements. No enticements or compensation for participation were provided.

### Procedure and data collection

Participants were recruited through institutional and personal social media (Facebook, Twitter and Instagram) accounts of the authors. The authors and their institutions were located in Australia, United Kingdom and New Zealand, and encouraged sharing of advertisements. Data were collected through a purpose-built survey collaboratively developed by the author team. The team consisted of five clinician researchers, two parents with no research experience and one footwear industry representative.

Data were collected online via Qualtrics® software (Qualtrics, Provo, UT, USA) [18]. The survey was developed in English; however, this software also allows users to have information displayed in their preferred language, The accuracy of translation was not tested, nor were we able to track how often this feature was used.

Participants were informed they could close and exit the survey at any time and any answers would be used within the final analysis. Participants were asked to self-select which stakeholder category there were aligned with and answered questions relating specifically to that category. For example, parents were asked the age of their youngest child, health professionals were asked what their profession was, and footwear industry or researchers were asked how long they had worked in that field. Participants were able to select more than one category and were presented with all relevant questions. All participants were also asked their gender and residing country.

**Fig. 1:**
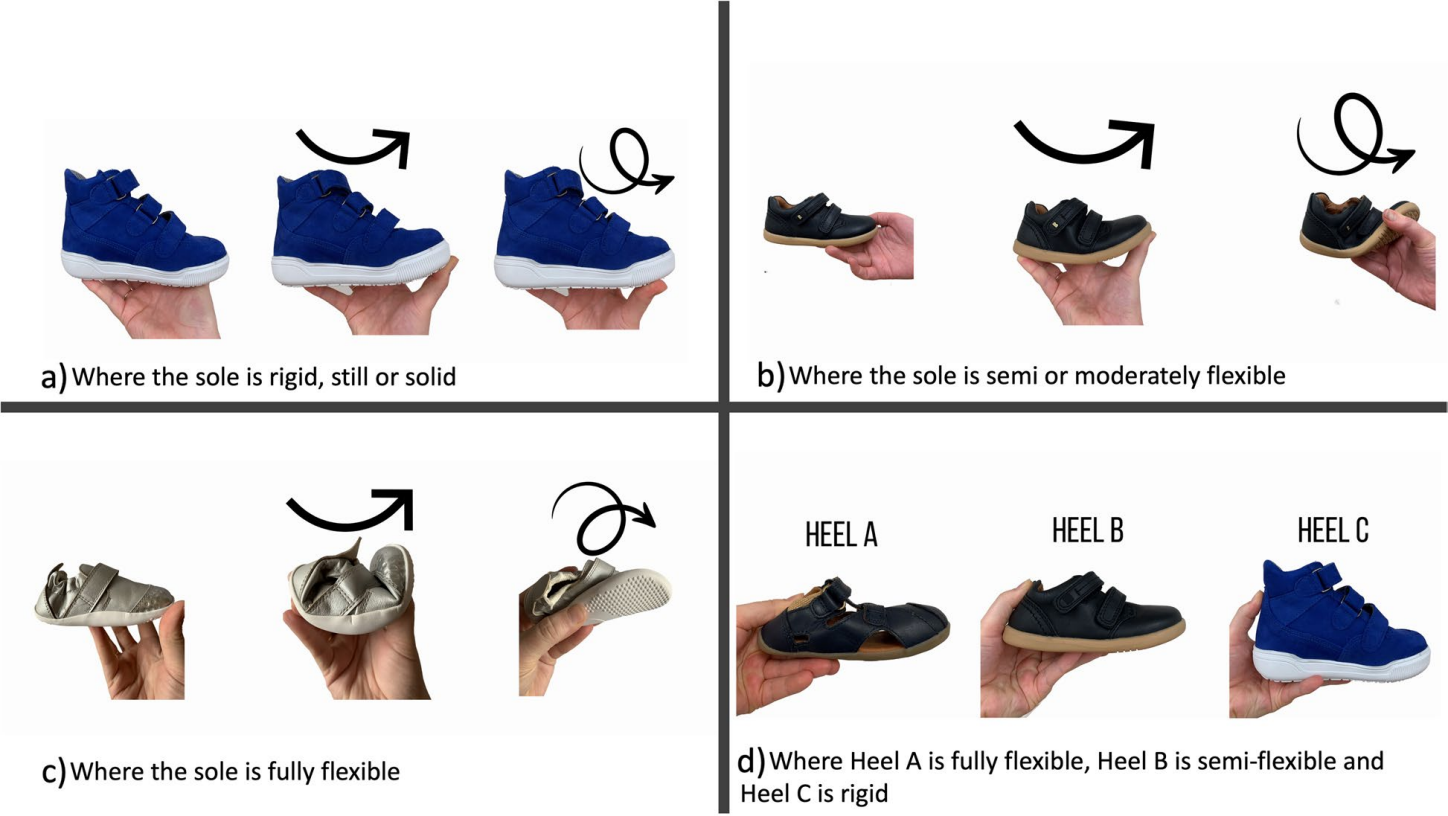
Pictorial prompt displaying different types of sole flexibility in bend and twist and heel counter flexibility

Participants were guided to think about differences in footwear sole and heel counter flexibility. There were 12 different pictures that prompted participants to consider footwear with very flexible, moderately flexible and limited flexibility at specific footwear structure points. Participants were asked the three following questions: “*If the flexibility of the sole is important to you, can you describe why?*”, “*If the flexibility of the heel counter is important to you, can you describe why?*” and “*If some, or any other footwear features are important to you, please describe why?*” (Fig. 1).

Cookies were used to allow responses to be saved up to 4 hours within partial completion. Qualtrics® routinely collects Internet Protocol (IP) addresses as part of the de-identified metadata in the survey response. These were not viewed as part of this research relating to this aim.

### Analysis

Data were initially cleaned, and responses removed if participant did not fully complete questions about which stakeholder group they belonged to. Data were analysed in Microsoft Excel 2018 (Microsoft Corp, Redmond Washington). Participants were grouped in a hierarchical manner based on health professional status. For example, responses from health professionals who were also parents, were grouped in the health professional category.

Inductive thematic analysis was undertaken. This method of analysis allowed for the short comments to be individually considered and used for theme generation [19]. Originally, comments were grouped against concepts and then during concept review, meaningful themes were developed. There was little variety in the statement length from participants. Even if the statement was short, it was grouped where possible. This grouping took an iterative approach, whereby if a new concept or theme developed, earlier statements were re-coded.

The data were initially analysed by a female researcher (CW). To reduce individual bias, themes and individual statements were independently reviewed by authors also aligned with the stakeholder category of their experience. Authors HB (female), KP (male), MH (male) and SM (male) reviewed health professionals’ statements, SB (male) reviewed footwear industry statements and KG (female) and EH (female) reviewed parent statements. Reflexivity was acknowledged as a concept introducing personal bias into research, discussed among the authors to minimise impact on reporting results [20]. Authors analysing qualitative data also acknowledged different individual experiences based on training and footwear experience, and how these different experiences may have influenced analysis.

## Results

### Participant characteristics

There were 159 participants who consented to complete the survey, 121 provided full demographic responses (Table 1). The health professional participant grouping included 42 podiatrists, 39 physiotherapists and 9 other health professionals (orthotists/pedorthists). Of these 90 health professionals, 54 (60%) had greater than 4 years of experience in prescribing or giving advice on footwear for young children. There were 55 (45% of 121) participants who had children under 6 years of age, and nine participants who worked in the footwear industry including footwear designers or those working in retail, four of these participants were also health professionals. The median (IQR) number of children of all participants was 2 (1, 2) and the median child age was 3 (1,4) years.

**Table 1:** Participant demographics

	Total participants (*N* = 120)
Country, n(%)	
*Australia*	65 (54%)
*United Kingdom*	30 (25%)
*USA*	11 (9%)
*Other*^a^	15 (12%)
Female, n(%)	98 (91%)
Health professionals, n (%)	
*Podiatrist*	42 (47%)
*Physiotherapist*	39 (32%)
*Orthotist/Pedorthist*	9 (10%)
Footwear industry stakeholders n (%)	
*Researcher*	1 (1%)
Footwear industry (design or sales)	9 (7%)
Parent of child/ren ≤6 years of age	54 (45%)

^a^Canada, Malta, Singapore, Denmark, New Zealand

**Fig. 2:**
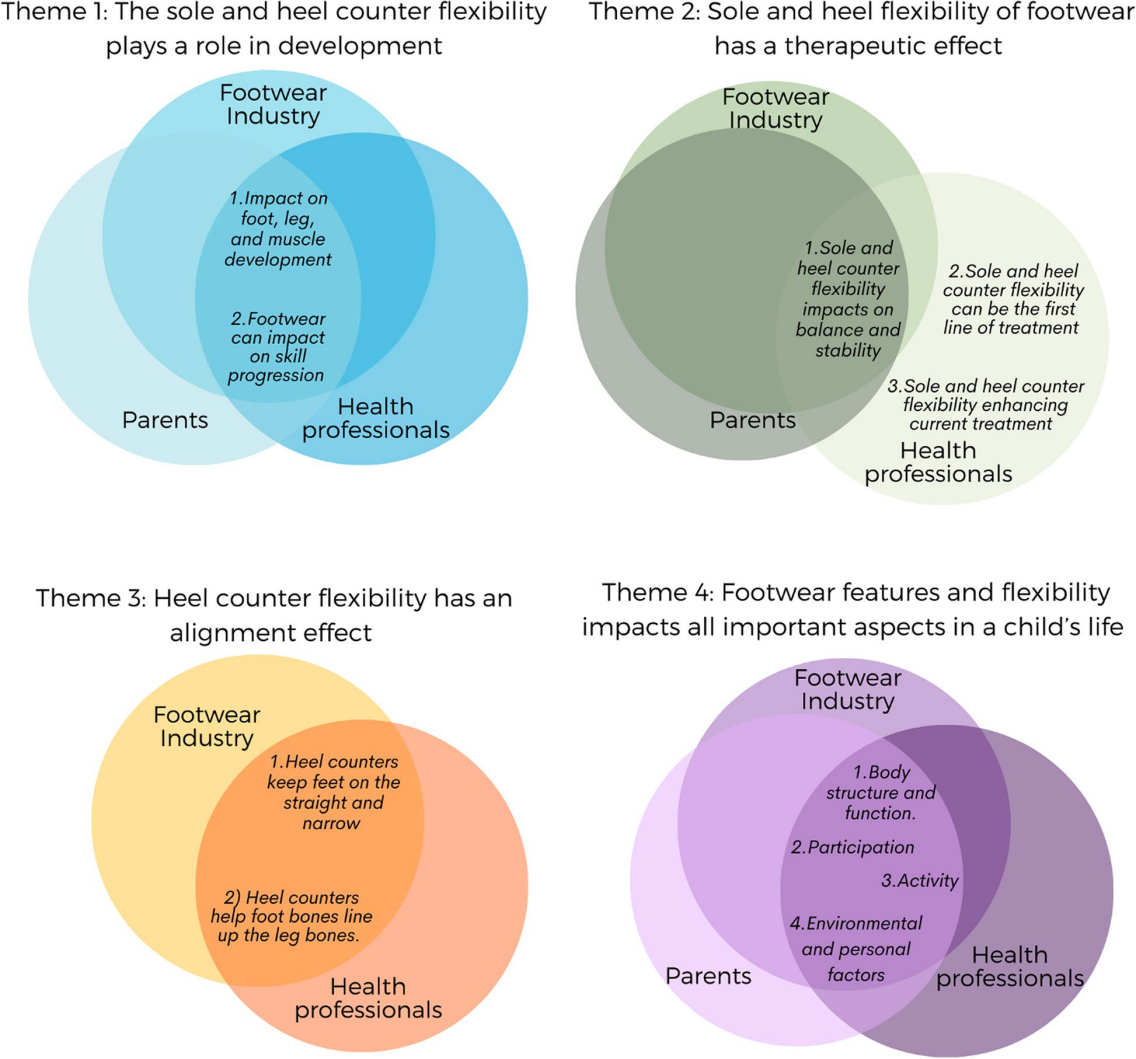
Four major themes and subordinate themes identified by stakeholder groups

We developed four main themes from the statements provided by the different stakeholder groups. These are illustrated in a map of themes, subordinate themes, and the stakeholder groups these themes aligned to (Fig. 2).

### Theme 1: the sole and heel counter flexibility plays a role in development

Participants described their beliefs of footwear sole and heel counter flexibility as being strongly linked with various aspects of the child’s development. There were two subordinate themes: 1) *Impact on foot, leg, and muscle development* and 2) *Footwear can impact on skill progression.*

All participant groups described beliefs relating to sole flexibility having an *Impact on foot, leg, and muscle development.* Comments ranged from parents describing flexibility or firmness impacting on foot growth such as “*Small children who are growing fast and more susceptible to their foot and foot shape being affected by footwear need really flexible shoes” (participant number (p) 78)* and health professionals describing the importance of flexibility of the sole supporting muscle control during different developmental stages such as “*[Shoe A] fully flexible important for non or new walkers to allow development of intrinsic ankle foot control, [Shoe B] flexible important for toddlers to age 2 or 3 for more foot protection outdoors while still allowing ankle foot control development” (p1).* Footwear industry stakeholders also considered sole flexibility levels as an important feature, stressing the need to get it right for each child and expressed concern over getting it wrong “*This is important to not interfere with their gait and not to cause stress on developing muscles” (p66).* At times, there was contradictory opinions expressed by participants about the impact of sole flexibility, an example from one parent being “*Foot develops well without shoes. I think more rigid soles are better” (p77).*

The other subordinate theme arose from feedback relating to how the flexibility at the sole and the heel counter was relating to ***footwear features can impact on skill progression****.* Participants in all groups expressed their strong beliefs about flexibility at these areas of the shoe and how this may differ over time. One parent highlighted this through their statement about sole flexibility with “*[Shoe A] is important for babies as you don’t want anything rigid, ideally most kids I think should feel the floor when they are young so that their feet adapt whilst they are learning to walk” (p83)*. Some health professionals described how heel counter flexibility could play a role in developing balance and stability during different developmental stages. One such statement was “*[Shoe] B is important for children to help them with coordination and balance with gait support” (p77),* when describing a moderately flexible sole and relating it to wear while a child was learning to walk. Participants in both the parent and health professional stakeholder groups also expressed uncertainty about the importance of heel counter flexibility, resulting in their difficulty to express exactly why flexibility at this area of the footwear was important. This was highlighted by one parent relating thoughts about both sole and heel counter flexibility as “*There are certain uses, and time & place for all [firmness] options” (p91),* and a podiatrist describing *“I am unsure on this. Have heard both scenarios that heel counter important for rear foot control but also more flexible heel counter is important to allow the natural foot motion. Unsure which to steer toward sometimes” (p107).*

### Theme 2 sole and heel flexibility of footwear has a therapeutic effect

There were three subordinate themes arising from participants relating the perceived therapeutic effect of footwear features including 1) *Sole and heel counter flexibility impacts on balance and stability,* 2) *Sole and heel counter flexibility can be the first line of treatment* and 3) *Sole and heel counter flexibility enhancing current treatment.*

All participant groups described beliefs surrounding s*ole and heel counter flexibility impacts on balance and stability.* Many participants described greater sole flexibility enhancing proprioception through thinner, more flexible sole materials. This was related to a flexible sole allowing greater sensory feedback from ground contact. Whereas others described the firmer sole giving stability when taking part in activities that would potentially challenge the child’s motor skills, such as long walks or walking over uneven surfaces. This was echoed by the footwear industry stakeholders through comments such as “*The thin sole allows the child to feel the ground which activates their pro-receptors [sic]” (p66)* and a physiotherapist, who provided the rationale that heel counter flexibility “*provides better stability at the heel. This may improve a child’s ability to balance...If foot moves inside shoe child may need to claw with their toes to hold foot still in shoe. Delays their balance response” (p28).*

Health professionals were the only participants who described footwear flexibility features within a treatment framework for the following two subsequent subordinate themes. For example, Sole and heel counter flexibility can be the first line of treatment was highlighted by an orthotist in relation to gait conditions with “*Stiffened sole to help with standing in low toned children” (p8)* and a physiotherapist described *“I often use shoes like picture C for children who walk on their toes to minimise their ability to do so” (p40).* Podiatrists commented on their beliefs in relationship to specific foot movements being impacted by footwear flexibility “*so the hallux can dorsiflex to allow windlass* [mechanism] *to take effect for effective gait” (p12) and* “*It can be the difference between needing an orthotic or not” (p45).*

Lastly, health professionals were also the only ones who related that the sole and heel counter flexibility enhancing current treatment when treating children who relied on assistive technology for mobility, or who had a foot deformity because of a disability. A physiotherapist described that “*a more flexible heel counter can make the shoe easier to get on and off, especially if the child uses an orthosis or prosthesis” (p3)* and a podiatrist highlighted *“Shoe C would be good with orthotics/AFOs and for toe walkers to control the amount of comfortable PF [plantarflexion]” (p40).*

### Theme 3 heel counter flexibility has an alignment effect

Health professionals and footwear industry stakeholders described beliefs relating to the heel counter flexibility (or firmness) having an alignment effect, while no parents provided any statements relating to this concept. Within Theme 2, health professionals and footwear industry stakeholders provided statements about flexibility being linked to a therapeutic goal, or as part of treatment interface between the footwear and an orthotic device. In this theme, the participants provided stand-alone quotes relating to the importance of footwear for visual alignment or a straighter appearance. There were two subordinate themes, primarily relating to the flexibility of the heel counter in having a proximal, local or distal impact. These were 1) *Heel counters keep feet straight* and 2) *Heel counters help foot bones line up the leg bones.*

*Heel counters keep feet straight* related to participants statements about the firmness of a heel counter being essential to straighten the appearance of the foot, exerting a distal effect on the whole foot. For example, a physiotherapist reported heel counter flexibility being important to “*To control the hind foot and subsequent mid and forefoot...control the heel and mid foot we control muscle and bony alignment” (p35).* This theory was supported by a podiatrist stating heel counter flexibility was important “... *as the rearfoot will support the midfoot and for each age” (p108).* A participant in the footwear industry stakeholder group also described this feature as important because the “*Heel counter holds the ankle bone up straight, preventing pronating. This is especially important in younger children as it can help guide the arch in the right direction and prevent the need for orthotics later in life” (p122).*

The subordinate theme relating to *heel counters help foot bones line up the leg bones* was described through statements such as “*they need to have important heel support to help them with their movements of walking, running and jumping. A firm counter at an age when they need the support is important for their alignment” [Podiatrist, p108],* and a physiotherapist describing heel counter flexibility as important “t*o aid in supporting ankle alignment” (p30).*

### Theme 4 footwear features and flexibility impacts all important aspects in a child’s life

All participants provided other comments on footwear features relating broadly to footwears’ impact on children’s lives. These comments instinctively aligned with the domains of the International Classification of Functioning, Disability and Health [21]. These being a) Body structure and functions, b) Participation and c) Activity and d) Environmental and personal factors.

All participants described footwear features and their potential impact on *body structure and functions.* The important footwear features described by podiatrists were in relation to footwear dimensions (e.g., length, width, depth) such as “*Many children have problems with toenails as they grow and I have frustratingly found that shoes just do not have enough depth at the toe box” (p101)* and “*The width of the shoe...A narrow foot in a wide shoe gets lateral motion causing the child to grip their toes to hold on. They generally don’t complain of sore feet, however the complain of sore shins. This is from the muscles in the toes and shins contracting. Obviously also a wider foot in a narrow shoe will create corns, and in the long run bunions” (p121).*

Whereas other participants in all groups described footwear features relating to *participation*, including important features of footwear that support independence in dressing, ease of donning and doffing by the parent, particularly when in a hurry, fixtures that prompted child independence for self-dressing and features that enabled footwear to be fit for desired purpose, such as waterproofing on boots for the snow.

Additionally, all participants provided statements supporting the flexibility of footwear features relating to enabling children to engage in age-appropriate *activity.* A footwear stakeholder described important sole features including “*Another one I would like to highlight - is shock absorption, something that I think there should be more of for children and not only for sports but in general” (p63),* and both parents and different health professionals related that comfort had a direct relationship with activity and the footwear feature flexibility.

Lastly, all participants also described flexibility footwear features relating to environmental and personal factors. These factors included the cost and durability relating to how flexible the footwear was. Parents also commented on the look of the footwear and how they perceived the practicality of the footwear would fit into the family lifestyle.

## Discussion

This study explored the perceptions of flexibility of footwear components and other features determined important by parents, health professionals, and footwear industry representatives. In doing so, we identified several differences relevant to stakeholder experience, whilst determining themes related to the impact footwear flexibility has on development, desired therapeutic effects, foot and/or leg alignment and the child’s life (including function and participation, activity and personal choice effects).

Encouragingly, as the first exploration of perceptions around footwear flexibility, participant groups frequently identified common outcomes associated with increased and decreased sole and heel counter stiffness. All stakeholders also expressed the belief that sole firmness needs to meet the developmental requirements of the child, which was noted to change over time and circumstance. Comments such as increased flexibility ‘allow for intrinsic ankle control’ and being important to ‘not interfere with gait’ when discussing new walkers, yet suggesting that increased stiffness improves ‘balance and stability’, cited as essential if the child’s motor function is being challenged. It has, however, also highlighted some inconsistencies in perceptions of what footwear flexibility can, or does, impact upon. Specifically, whilst parents, health professionals, and footwear industry representatives all described limited heel counter flexibility was associated with development, potential desired therapeutic effects, and impact on the child’s life, only health professionals and footwear industry people were concerned regarding the potential impact on foot alignment. Both groups identified a firmer heel counter as more likely to result in straighter feet, with concern expressed for more distal structures if the rearfoot was not stabilised. This most likely reflects the education and focus of these different professional groups, however, is not universally supported by evidence. For example, one participant went as far as to link a firmer heel counter with improved arch development and a reduced need for orthoses later in life. There is no known empirical research that indicates footwear can improve foot development or structural features of foot shape, such as the arch height in children [9]. Conversely, evidence suggests footwear may actually have an opposite effect [22], this further highlights the need for footwear recommendations to be evidence-based.

Evidence of how footwear flexibility impacts on the developing foot, leg and/or muscle function is an emerging area with many of our common recommendations based on observations of adult-based studies [23, 24], or potentially, on folklore which has been ‘accepted’ over time. Recently, there has been an increasing research focus on understanding the impact of sole flexibility on gait and foot function in younger children to address this evidence deficit. This has included findings of limited impact on toddler gait with very flexible soles [13], changes in young children’s walking and running in different types of footwear between barefoot and soft soles [25], and minimal differences in gait and foot function between medium to firm soled footwear in preschool and school aged children [26].

The findings of this qualitative study need to be viewed in the context of the limitations and strengths of participant representation and methodology. Firstly, this study was open to anyone who met the criteria, but uptake was primarily from health professionals, with only limited representation from footwear stakeholders and a smaller number of parent-only participants. There is inherent responder bias based on the interest of participants in footwear, and the survey design in English. This limitation may have prohibited participants from non-English speaking countries where parents may hold different opinions and values about children’s footwear features. The impact of this is on the study outcomes are unknown. Conversely, the strength of this research lies in its co-design with parents and footwear industry representation within the research team, the strong international participant responses which improves the global context of our understanding. Furthermore, it is the first exploration of its kind known to the author team, to involve such broad stakeholder groups. As stated earlier, footwear recommendations are plentiful, however predominantly developed by those involved in footwear design and sales, where there is motivation to have the design specifics of their product seen as important. Furthermore, the rise of social media ‘influencers’ has increased the capacity for people, potentially without adequate knowledge on factors affecting developing bone and soft tissue structures, to have a stronger impact than ever before. As such, many footwear recommendations may be subject to trend rather than based on sound concepts. Foundational children’s footwear research data fields have recently been formed through a consensus-based taxonomy of children’s footwear styles and features, establishing recommendations on defining therapeutic footwear, footwear design characteristics and prescription variables [15, 16]. The next steps for children’s footwear research must involve developing a consistent suite of outcome measures to add to these foundation data fields. The use of these fields should then be used in any children’s footwear research to assist in challenging or confirming stakeholder views. Use of foundational data in this way will allow for combining studies to improve the strength of footwear recommendations for children of all abilities.

## Conclusion

This work adds to the growing body of knowledge on footwear characteristics. This research also helps health professionals and footwear stakeholders to understand where footwear messages can be improved. This is important to minimise misinformation about children’s footwear, benefits, and harms. The findings from this research should prompt health professionals and footwear stakeholders to consider how messaging impacts unfounded or evidence supported beliefs about footwear features. This information is key to developing contemporary footwear recommendations for parents and caregivers. Having recommendations and appropriately developed resources is essential to support parents and caregivers’ footwear decisions for their child.

## Data Availability

All available data is provided within the manuscript.
